# Empowering human-AI teams via Intentional Behavioral Synchrony

**DOI:** 10.3389/fnrgo.2023.1181827

**Published:** 2023-06-20

**Authors:** Mohammad Y. M. Naser, Sylvia Bhattacharya

**Affiliations:** ^1^The Neuro-Interaction Innovation Lab, Kennesaw State University, Department of Electrical Engineering, Marietta, GA, United States; ^2^The Neuro-Interaction Innovation Lab, Kennesaw State University, Department of Engineering Technology, Marietta, GA, United States

**Keywords:** human-AI teaming, Intentional Behavioral Synchrony (IBS), human-machine trust, Human-Machine Interaction (HMI), multimodal fusion

## Abstract

As Artificial Intelligence (AI) proliferates across various sectors such as healthcare, transportation, energy, and military applications, the collaboration between human-AI teams is becoming increasingly critical. Understanding the interrelationships between system elements - humans and AI - is vital to achieving the best outcomes within individual team members' capabilities. This is also crucial in designing better AI algorithms and finding favored scenarios for joint AI-human missions that capitalize on the unique capabilities of both elements. In this conceptual study, we introduce Intentional Behavioral Synchrony (IBS) as a synchronization mechanism between humans and AI to set up a trusting relationship without compromising mission goals. IBS aims to create a sense of similarity between AI decisions and human expectations, drawing on psychological concepts that can be integrated into AI algorithms. We also discuss the potential of using multimodal fusion to set up a feedback loop between the two partners. Our aim with this work is to start a research trend centered on exploring innovative ways of deploying synchrony between teams of non-human members. Our goal is to foster a better sense of collaboration and trust between humans and AI, resulting in more effective joint missions.

## 1. Introduction

In coherence with the rapid growth of interest in Artificial Intelligence (AI) applications, it is inevitable that AI will play a vital part in humans' lives (Haenlein and Kaplan, 2019). As a result, the upcoming years will see various forms of human-machine teaming across several domains and applications, including those involved in high-sensitivity and complex tasks, such as military applications (National Intelligence Council, 2021), transportation (National Science and Technology Council United States Department of Transportation, 2020), and others. Studying the dynamics of human-AI teams is an interdisciplinary endeavor that involves technical concepts related to the intelligent part (such as computer science and engineering) and the human element (such as psychology). It also entails examining the interactions between humans and AI, which in addition to the concepts above, encompass a wide range of considerations, including human factors, ethics, and policymaking. This area of research is becoming increasingly important because fewer studies are available that examine the interactions between humans and AI than those that focus solely on the individual elements. Thus, there is a growing interest in understanding how humans and AI can work together in a way that is safe, ethical, and effective.

Although AI is a new field, research into it does not have to begin from scratch. Rather, we view AI as a subdomain of the machine-based automation movement that started in the mid-1900s. Therefore, there is a wealth of research and knowledge that we can use to design better human-AI systems. Furthermore, by viewing AI as an evolved, more-sophisticated version of a rule-based machine or a robot, one of the key pillars in understanding the relationship between humans and AI is human trust, a topic that has been addressed by many over the years, of those which date back to the 1980s (Muir, 1987). From the literature, we define human trust in AI as the degree to which a user holds a positive attitude toward an AI team member's reliability, predictability, and dependability to perform the actions that contribute to delivering the overall task aims. Trust establishment is influenced by many factors, which can be divided into three categories: human factors (about humans), partner factors (about AI), and environmental factors (about context), all of which are interconnected (Hancock et al., 2011). Achieving the correct balance and alignment between human trust and AI capabilities requires accounting for all these factors, a concept known as “calibrated trust” (Lee and See, 2004). This work follows in the same vein by introducing a new concept that has the potential to help enhance our knowledge of human-AI team systems.

In a human-human team setting, the level of trust the trustor places in the trustee is influenced by the latter's ability to defend themselves and explain the rationale behind their decisions. This is also extendable to human-AI teams (Chen et al., 2016). However, this is a challenging feature to have in AI (Calhoun et al., 2019). Thus, instead of imposing the explainability load on AI, we propose shifting this burden to humans by having AI imitate their actions in low-stakes subtasks. By recognizing themselves in the actions of AI, humans can set up a connection with it. As a result, in decision-making instances where the best decision is unclear, humans can trust the AI's decision based on the pre-established trust from earlier, more direct decision-making interactions. We are calling this model “Intentional Behavioral Synchrony (IBS),” and we define it as the intentional adoption of minor decision-making patterns by an AI agent to mimic those of its human teammate in scenarios where such decisions have a minimally noticeable impact on the mission's overall aims. This approach aims to enhance human-AI teaming, improving team performance in the long term. This paper aims to introduce this concept and discuss its origin and ability to implement it in real-life applications.

## 2. Intentional behavioral synchrony

### 2.1. Theoretical foundation

The IBS concept draws inspiration from a psychological phenomenon seen in human-human interaction, known as interpersonal synchrony. Traditionally, interpersonal synchrony refers to physical and temporal coordination (Schmidt et al., 1990), but it has been found to involve several other behavioral mechanisms beyond movement (Mazzurega et al., 2011). It is well-established that synchrony is an indicator of social closeness between two humans (Tickle-Degnen and Rosenthal, 1987). This concept can be easily extrapolated to human-agent teams, but only for agents with similar or very closely matched behavioral and cognitive abilities to humans. But here we are concerned with AI-powered machines, or agents, that have the abilities of processing complex-sets of information, reasoning, decision-making, and taking actions, in pursuit of general high-level aims [definition is inspired by Gabbay et al. (1998)]. These agents are not necessarily humanoid, and they might be embedded in a piece of software that is invisible to humans. This is why synchrony, in the traditional sense, cannot be used with such systems. Thus, the IBS concept was introduced. IBS can be thought of as an add-on to currently existing AI elements of different nature. It is a trust-building strategy for humans working with AI teammates, which can be integrated into AI algorithms to enhance the team's overall performance.

The importance of trust comes from its being essential in building a personal relationship or companionship with a teammate. This human trait has been shown to advance team efforts in achieving their goals (Love et al., 2021). Humans strive to prove a sense of companionship with their possessions, as shown by many individuals naming their belongings. This practice is prevalent across various domains, including sensitive environments like the military, where equipment is often named, such as the famous Big Dog robot. Therefore, by implementing IBS in AI algorithms, we can create a sense of harmony that aligns with human expectations, thereby fostering stronger connections between team members, allowing humans to trust more sophisticated decisions made by the AI that humans may not be able to attest the optimality of the decisions made.

### 2.2. Operational environments

The proposed strategy is fundamental in nature and, thus, can be implemented in a range of scenarios. Here, we offer some guidelines to illustrate how IBS can be effectively used.

#### 2.2.1. Not limited to robots

IBS is meant to supply a connection between a decision-making element and a companion human to ease joint task performance. This connection is not restricted to robots or humanoid agents but can extend to any type of intelligent entity. For example, it could be a software application making actions, whether visibly or invisibly, that affect the joint task. Also, IBS and other behavioral synchrony mechanisms are expected to work effectively with humanoid robots, which may have greater potential than invisible AI agents.

#### 2.2.2. Not limited to specific types of interaction

Regardless of the freedom given to AI and whether its role is complementary to humans in making joint decisions or if it handles making independent decisions, the trust value brought by behavioral synchrony is unquestionable. So, IBS can be used in various applications regardless of the AI's interaction with the human partner. Furthermore, the significance of trust between humans and AI is not limited to specific domains where the human's emotional state is crucial to achieving team aims but is a fundamental aspect of teamwork. Hence, it is essential to consider trust when perfecting the design of AI machines to maximize the overall benefits generated by the team.

#### 2.2.3. A personalized fashion

Each human has a unique perspective and set of qualities that affect their ability to work effectively in teams and interact with AI, which is referred to as dispositional trust in literature (Hoff and Bashir, 2015). Dispositional trust develops from human characteristics, such as accumulated experiences and life events met by the individual up to the present time. These interactions significantly affect human behavior and can leave individuals vulnerable to intentional or unintentional biases, which can affect how they collaborate with AI in joint systems. As a result, IBS integration, as well as any trust-building mechanism, will need to be conducted on an individual basis. Nonetheless, clustering techniques could be used to find shared characteristics among humans that could ease the process.

## 3. Practical framework

### 3.1. Description

**Figure 1:**
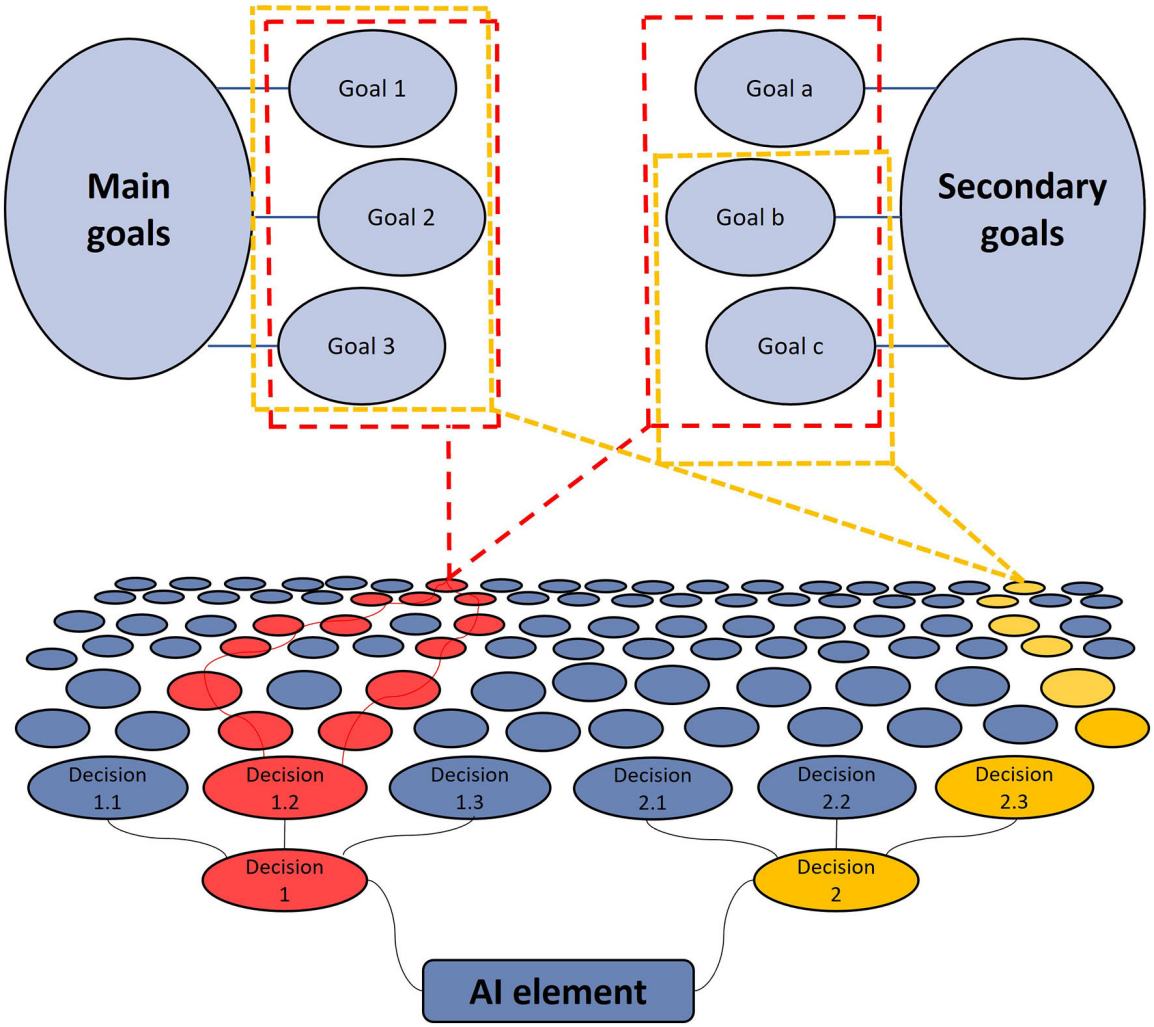
An exemplary diagram of AI decisions in a complex multi-layer mission.

Our vision is to use IBS and behavioral synchrony in highly dynamic and complex environments where tasks are layered with multiple goals and aims. These environments offer many opportunities to test the effectiveness of our theory in real-world applications; they have the freedom to try alternative approaches (decisions) that eventually can achieve the same goals. To give a perspective on how such a strategy would work, consider the exemplary diagram in Figure 1. The figure illustrates how an AI element is positioned to make decisions in a hierarchical manner, where each decision affects the later set of decisions. These decisions are interdependent and driven by a set of primary and secondary goals that AI has been programmed to achieve. In the figure, AI is one of many elements of a network of elements involving the human companion and other AI-human teams. This setup is ideal for applying IBS and similar algorithms.

At each step of the decision-making process, the decision that contributes to achieving the greatest number of goals is preferred. This is how intelligent elements should be programmed. In the figure, these are the paths shown in red. In such an environment, AI can carry out the goals set by humans in diverse ways by following one of the red routes. Thus, no goals or aims of the experiment will be compromised by taking either path. The proposed IBS method suggests prioritizing the path that is more like what a human would choose, not just at the first stage but throughout the decision-making process. This can be implemented by adding this IBS requirement to the list of secondary or even tertiary requirements of the systems. While this is not an aim of the specific mission itself, it aims to build a connection with the humans involved which may help achieve other strategic goals eventually, using the AI algorithms developed in this training mission.

However, certain decisions made by AI could compromise the delivery of all aims, as depicted in the yellow path in the graph. In the case that the yellow path is much more intuitive to humans compared to the red paths, the challenge becomes about making comparisons and analyzing trade-offs between specific task aims (achieving as many goals as possible) and overall strategic aims (building robust AI algorithms that work efficiently and effectively with humans). The challenge lies in analyzing the associated risks and rewards for interconnected teams composed of many elements. There are two ways of integrating IBS to address situations where adding IBS criteria may compromise the quality of work. The first method involves integrating this element during the training phase in an offline manner. Once sufficient training has been completed, humans should have enough confidence in the AI's reasoning abilities to make proper decisions without the need for real-time IBS. The second approach is to keep IBS implemented during actual task execution to supply a continuous real-time sense of similarity to the human, simply by avoiding actions that are out of the ordinary.

### 3.2. Human reflections for feedback

To test the effect of AI decisions on humans and whether that is the ideal alternative from a set of options or not, one potential measurement method is the use of self-reported questionnaires. However, this method is susceptible to biases such as recall, interpretation, and social desirability biases, among others. An alternative approach is incorporating real-time human signals into the AI system. The use of physiological modalities in Human-Machine Interaction (HMI) applications is prevalent. In settings where signal acquisition devices are available, they could be used to set up a feedback loop from humans to the AI system to evaluate its decisions. However, this strategy has added constraints, most notably signal interpretation and the necessity to isolate the outside events from the targeted events with the machine. These obstacles may be outweighed by the insights physiological signals provide that cannot be gained through other traditional approaches.

Different human physiological signals have been long set up to be correct in detecting human emotions, including trust as one such emotion. Because of established neural correlates of trust, Electroencephalography (EEG) has been one of the key modalities used to extract trust levels (Long et al., 2012). The earliest attempts were mostly confined to using Event-Related Potentials (ERPs) to extract the brain response of humans while completing specific cognitive activities. Studies of this sort include (Boudreau et al., 2009; Long et al., 2012; Dong et al., 2015). The ERP (Event Related Potentials) approach is hard to be integrated into practical systems, and thus we would not recommend it. Furthermore, in a study published in Akash et al. (2018), EEG and Galvanic Skin Response (GSR) were used to find links with trust. The authors were successful in doing so, with accuracy values in the 70% range. GSR was also shown to correlate with trust levels in studies of different contexts, such as the work of Montague et al. (2014) and Khawaji et al. (2015). Furthermore, there have been several recent studies examining EEG for detecting the neural markers of human trust, such as Wang et al. (2018), Blais et al. (2019), Jung et al. (2019), and Oh et al. (2020). Electrocardiography (ECG) is far less researched compared to EEG and GSR. We are not aware of any studies that examined implementing ECG for capturing human trust in the context of intelligent machines. With that, some studies have shown a relationship between heart rate and other ECG features with trust in contexts not related to HMI, such as the work shown by Leichtenstern et al. (2011) and Ibáñez et al. (2016). Also, there are a few studies that examined the mapping between eye-tracking with trust, but in applications different from the application of interest here.

In addition to the physiological signals, there is a prospect of using facial expressions to detect trust and other emotions of the human in such systems. Using deep neural networks trained on video footage, which is more prevalent and easier to implement than certain physiological modalities, can classify human emotions. Detecting human emotional states from facial expressions is a broad area of research, and it is well-established to be amazingly effective (Ko, 2018; Naga et al., 2021).

As shown above, many inputs from humans have shown a potential in detecting human trust. Also, some studies have been undertaken to investigate the validity of multimodal fusion techniques in detecting human trust and emotions, such as Zheng et al. (2014), Huang et al. (2017), and Zhao et al. (2019). That said, multimodal fusion is still in the first stages of development. Still, it holds the potential for creating robust classification schemes, leaving room for further research on integrating different physiological modalities to quantify the trust between humans and AI.

### 3.3. Supporting evidence

By examining the relevant literature, one can investigate the soundness of the proposed approach. Here, two fundamental questions are discussed.

#### 3.3.1. Can familiarity/similarity help establish trust between humans and AI?

Explainability is crucial for building trust with machines, but it is not always feasible. To address this, IBS is introduced as a tool to create similarity. Our aim is to replace the need for explainability with similarity, supported by the work of Brennen (2020) where users were found to build trust in AI through familiarity rather than detailed explanations of its working methodology. Furthermore, establishing trust based on behavioral imitation is a well-known psychological human trait. In the work of Over et al. (2013), it was demonstrated that children place great importance on establishing trust with adults when the adults imitate their choices. Other studies have also examined the importance of shared traits in establishing trust. For instance, in the study of Clerke and Heerey (2021), higher levels of behavioral mimicry and similarity were shown to lead to greater trust between individuals, which further supports the validity of exploring the IBS method for establishing trust in human-AI systems.

#### 3.3.2. Is it possible to have the same/similar output despite making different early decisions?

In simple applications of singular goals, such as in a chess game, it is indeed possible to obtain the same or similar outcome despite making different early decisions (Sutton and Barto, 1998); different openings can lead to the same output of winning. However, when a network of sub-goals is introduced, such as aiming to finish the game in the shortest time, the process becomes more complex. While concrete empirical evidence is necessary to provide a definitive answer, it is reasonable to expect similar outputs, regardless of the early decisions made. This is because the number of possible paths (decisions) is highly likely to surpass the number of potential outcomes. As a result, some of these paths will ultimately lead to very similar outputs, providing many options for the machine to learn while establishing a relationship with the human partner.

## 4. Conclusions

In this brief conceptual study, we have introduced the idea of Intentional Behavioral Synchrony (IBS) as an essential step toward building trust between humans and AI machines in joint systems. We have highlighted the significance and positive impact of synchrony between humans and AI elements while working together as a team, especially in highly dynamic and complex tasks. Although our study does not supply empirical evidence, it offers valuable insights for examining the impact of IBS in real systems and encourages further research to explore factors that have been overlooked or underestimated in setting up more effective human-AI teams. Future studies could include investigating different mechanisms for measuring human reactions to AI in such dynamic applications. Also, the work paves the way for innovative ways of achieving synchrony among teams that include non-human members. Such an endeavor requires the collaborative effort of a multidisciplinary team of engineers, data scientists, and psychologists and must be executed in a context-dependent way.

## Data availability statement

The original contributions presented in the study are included in the article/supplementary material, further inquiries can be directed to the corresponding author.

## Author contributions

MN conceived and authored the entire paper under the direction of SB. All authors contributed to the article and approved the submitted version.
